# Multiple Intestinal Intussusceptions as a Complication of Severe Hyperglycemia in a Patient with Diabetic Ketoacidosis

**DOI:** 10.1155/2012/526041

**Published:** 2012-09-26

**Authors:** Pooja Raghavan, Jeffrey Salon, Dhyan Rajan

**Affiliations:** ^1^Department of Medicine, Medical Education, Mount Carmel Health, 793 West State Street, Columbus, OH 43222, USA; ^2^Division of Critical Care Medicine, Mount Carmel Health, Columbus, OH 43222, USA; ^3^Department of Medicine, Nassau University Medical Center, East Meadow, NY 11554, USA

## Abstract

Intussusception in adults is a rare phenomenon, occurring in approximately 1 in 30,000 hospital admissions annually. When it does occur, the majority of cases involve an organic lesion serving as a lead point for intussusception, such as tumors or postoperative adhesions. In a small percentage of cases, a lead point is not found, and intussusception is thought to be idiopathic or secondary to a disease process contributing to dysrhythmic peristalsis of the gastrointestinal tract. A few cases of functional intussusception have been reported as being secondary to severe hyperglycemia and metabolic derangements, including metabolic acidosis and hyperkalemia, by causing impaired gastrointestinal motility. We present a case of a 23-year-old Caucasian male who presented with severe hyperglycemia and diabetic ketoacidosis. Imaging of the abdomen revealed three intussusceptions involving the small intestine, which were easily reduced manually during exploratory laparotomy.

## 1. Introduction

Intussusception is a phenomenon more common in the pediatric population and is usually idiopathic or secondary to a viral etiology. Intussusception in adults is infrequent and typically occurs in the setting of an organic lesion serving as a lead point for intestinal telescoping, such as a tumor or adhesions from previous abdominal surgery [1]. Functional intussusception, occurring in the setting of a metabolic derangement such as severe hyperglycemia, metabolic acidosis, or hyperkalemia, is rare [2]. It has been suggested that severe hyperglycemia decreases gastrointestinal motility; GI dysmotility in turn has been implicated in the development of functional intussusception [1, 2]. Patients presenting with acute intussusception typically complain of severe diffuse abdominal pain, and the mainstay of therapy is surgical intervention.

A large percentage of patients who present with diabetic ketoacidosis complain of severe abdominal pain, and, in up to 75 percent of cases, the pain may be severe enough to be confused with acute abdominal pathology. These symptoms of abdominal pain are similar to the clinical presentation of intussusception. If intussusception is not detected or treated, it can result in severe complications, such as bowel necrosis, perforation, and peritonitis [2, 3]. We present a case of a 23-year-old Caucasian male who was noted to have 3 small bowel intussusceptions in the setting of severe hyperglycemia and diabetic ketoacidosis.

## 2. Case Presentation

A 23-year-old Caucasian male with a medical history significant only for insulin-dependent diabetes mellitus for 11 years was brought to the emergency department after his mother found him to be lethargic and confused that morning. History was difficult to obtain from the patient due to somnolence and confusion. As per the patient's mother, the patient had been in good health recently, with no recent infections or complaints of abdominal pain, fevers, chills, chest pain, shortness of breath, cough, sputum production, diarrhea, or constipation. The patient did not smoke, consume alcohol, or use illicit drugs. He had no surgical history, and family history was noncontributory. He had no recent changes in his medication regimen, which included insulin detemir 30 units subcutaneously every morning; insulin detemir 30 units subcutaneously every night; insulin aspart 4 units subcutaneously with breakfast, lunch, and dinner. The patient's mother was unsure if he had taken his basal dose of insulin the previous night or that morning. The patient's mother stated the patient had been compliant with medications in the past; however, due to recent financial struggles, he had not been taking his insulin regularly.

Physical examination revealed an ill-appearing male in moderate distress. The patient appeared tachypneic, with a respiratory rate of 16 breaths per minute. He was afebrile, with a blood pressure of 91/55 and a heart rate of 112 beats per minute. He was somnolent but was able to answer questions and follow simple commands. A complete neurologic examination was difficult to illicit; however, there appeared to be no focal deficits or signs of meningeal irritation. Mucous membranes were dry, and he had decreased skin turgor. Abdominal exam revealed a soft abdomen that was diffusely tender to palpation in all four quadrants. There was no rebound tenderness or guarding, and bowel sounds were appreciable.

Laboratory evaluation revealed an unremarkable complete blood count; blood cultures and urine cultures showed no growth. Arterial blood gas was suggestive of a severe metabolic acidosis, with a pH of 6.860 (reference range: 7.350–7.450), PCO_2_ of 9.8 (32–48), and HCO_3_ of 1.7 (21–28). Serum ketones were present, and a complete metabolic profile revealed severe metabolic derangements consistent with diabetic ketoacidosis (Table 1).

Central venous access was attained, and isotonic saline was administered as a 2 liter bolus, then at 250 milliliters per hour continuously. Simultaneously, regular insulin was administered intravenously as a bolus of 12 units and then continued at a rate of 3 units per hour. The patient was admitted to the intensive care unit for continued monitoring and treatment of severe hyperglycemia and diabetic ketoacidosis. With continuous infusion of intravenous fluids and insulin, the patient's severe hyperglycemia and diabetic ketoacidosis resolved after 18 hours. The patient now appeared more alert; however, remained in moderate distress. When asked, the patient stated that he had severe abdominal pain described as sharp, diffuse, and unremitting. Upon further questioning, the patient admitted to having abdominal pain this morning when brought to the emergency room, and the pain had been persistent during his hospital stay. Abdominal examination revealed a diffusely tender abdomen, with guarding present. There was no rebound tenderness; however, bowel sounds were now absent. 

**Figure 1:**
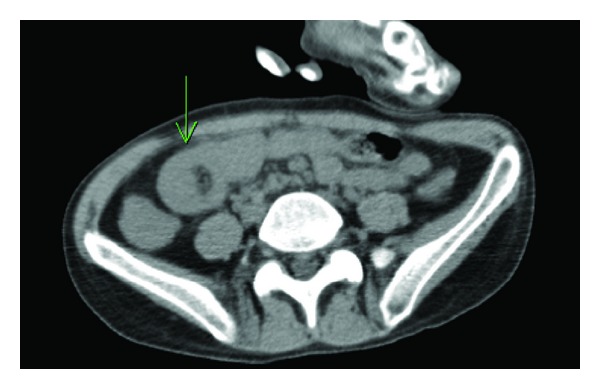
Computed tomography of the abdomen and pelvis revealing concentric, alternating layers of hyperechoic and hypoechoic shadowing within the intestine, suggestive of a duodenojejunal intussusception (arrow).

Given the patient's complaints of severe abdominal pain, a computed tomography (CT) of the abdomen and pelvis without contrast was performed. CT revealed three separate intussusceptions of the small bowel; the most proximal intussusception was duodenojejunal (Figure 1), and the two distal intussusceptions appeared to be ileoileal and ileocecal in nature. Due to the finding of three intussusceptions on imaging in conjunction with severe unremitting abdominal pain, the patient was urgently taken to the operating room for an exploratory laparotomy.

During exploratory laparotomy, the small intestine was examined in its entirety, and 3 areas of intussusception were identified: the first being duodenojejunal, the second ileoileal, and the third ileocecal. The small intestine looked healthy and viable, with no areas of necrosis. There were no physiologic lead points, abnormal lesions, or tumors identified in the small intestine. The rest of the abdomen was also explored, including the colon, stomach, and liver, which revealed no abnormalities. The three areas of intussusception were easily reduced manually in the operating room, and the patient's abdomen was closed. Shortly after the procedure, the patient admitted to complete resolution of the abdominal pain and now appeared well and in no acute distress. He was discharged home in stable condition on his usual dose of insulin detemir and aspart and educated on compliance of insulin usage. 

## 3. Discussion

Intussusception in adults is rare, occurring in approximately 1 in 30,000 hospital admissions annually [1]. The incidence of intussusception is nearly 20-fold more frequent in the pediatric population, with occurrence in most cases being idiopathic in nature or secondary to a viral etiology [1]. In nearly 70–90 percent of all adult intussusceptions, the presence of an organic lesion serving as a lead point that alters normal bowel peristalsis is found. These intraluminal lesions may be malignant such as adenocarcinomas, lymphomas, or metastatic disease or may be benign such as lipomas, hamartomas, or adhesions from prior abdominal surgeries [1, 2]. In 10 percent of adult intussusceptions, an organic lesion serving as a lead point is not found, and intussusception is thought to be idiopathic, or secondary to a disease process contributing to dysrhythmic peristalsis of the gastrointestinal tract [3]. Gastrointestinal diseases such as celiac disease and Crohn's disease have been reported to cause intussusception in the adult population; however, these occurrences are infrequent [3]. Metabolic disturbances such as hyperkalemia, metabolic acidosis, and hyperglycemia have been correlated with gastrointestinal dysmotility; however, these disturbances rarely result in bowel intussusception [3–5]. 

Both acute and chronic hyperglycemia can alter normal function and motility in every region of the gastrointestinal tract [4]. The most widely studied and well-known effect of hyperglycemia on the gastrointestinal tract is its role in gastroparesis, a condition reported to be prevalent in 20 to 55 percent of patients with type 1 diabetes mellitus and in 30 percent of patients with type 2 diabetes mellitus [6, 7]. Although the specific mechanism by which diabetic gastroparesis occurs is unclear, it is postulated that autonomic neuropathy involving the parasympathetic nervous system may lead to gastric dysrhythmias, a decrease in gastric tone, and alteration in antral size and motor function [8, 9]. These disturbances can lead to delayed gastric emptying, resulting in adverse symptomatology in patients with both acute and chronic hyperglycemia. Although the effects of hyperglycemia on gastric function appear to be well recognized as a potential complication in patients with diabetes mellitus, small intestinal dysmotility can also occur in individuals with hyperglycemia, potentially resulting in increased morbidity.

Small bowel motility appears to be frequently altered in patients with poor glycemic control, with several studies indicating impaired intestinal motility in the setting of acute hyperglycemia [5, 10]. In a study by Byrne et al. euglycemic and hyperglycemic clamps were utilized in order to determine the effects on fed jejunal motility in relation to varying glycemic states. The study concluded that acute hyperglycemia resulted in markedly delayed jejunal transit time, along with a reduction in amplitude of jejunal contractions illustrated via intestinal manometry [11, 12]. Russo et al. suggest a statistically significant reduction in the frequency of both duodenal and jejunal pressure waves in patients with induced hyperglycemia, along with decreased duodenocecal transit time during hyperglycemia when compared to individuals with euglycemia [4]. Hyperglycemia affects the central nervous system, myenteric plexus, and release of hormones from smooth muscle, which may collectively have an effect on normal bowel peristalsis [4]. Samsom et al. suggest that the pharmacologically induced inhibition of small bowel motility decreases the absorption of glucose from the lumen of the small intestine [13]. This may suggest that decreased intestinal motility secondary to hyperglycemia may serve as a physiologic compensatory measure to decrease glucose absorption and prevent further worsening of hyperglycemia [10, 13]. Intestinal dysmotility from hyperglycemia may result in abdominal pain, constipation, and intractable and recurrent vomiting. Rarely does hyperglycemia result in a functional bowel intussusception [4]. 

In children, the symptomatology associated with intussusception is often acute, resulting in a triad of abdominal pain, a palpable abdominal mass, and bloody, mucus-like bowel movements often described to resemble the appearance of “currant jelly” [14]. In adults, however, intussusception can result in a variety of symptoms which may be acute, intermittent, or chronic in nature [2]. In a retrospective study of 58 adults with a diagnosis of intussusception, Azar and Berger report the predominant presenting symptom to have been abdominal pain and nausea, occurring in over 75 percent of these individuals [2]. Diarrhea, constipation, gastrointestinal hemorrhage, and the presence of an abdominal mass were reported in some patients; however, the occurrence of such signs and symptoms was infrequent. The often variable and nonspecific complaints associated with adult intussusception can pose a clinical dilemma for physicians, especially in the setting of hyperglycemia and diabetic ketoacidosis (DKA). Nausea, vomiting, and abdominal pain are often present in patients with hyperglycemia and acidosis, symptoms similar to those with intussusception. Furthermore, it is believed that abdominal pain, which is directly proportional to the severity of acidosis, may be severe enough to be confused with an acute abdominal pathology in up to 75 percent of all cases of patients presenting with DKA [15–17]. Given the similarity of gastrointestinal symptoms seen in both intussusception and DKA, clinicians should have a heighted suspicion of intussusception in this patient population, especially when faced with disproportionate or unremitting abdominal pain. 

If adult intussusception is suspected, CT of the abdomen is currently considered the best diagnostic modality [18, 19]. A pathognomonic “target mass” which describes concentric, alternating layers of hyperechoic and hypoechoic shadowing within the intestine, should give rise to the suspicion of an intussusception [18]. Ultrasonography has proven to be beneficial in adult intussusception as it allows visualization of all planes of the abdominal wall and allows for a timely diagnosis when more sensitive modalities such as CT are not readily available [18]. Barium studies, such as a contrast enema, are of value in the diagnosis of colonic intussusception in adults; however, this study does not exclude small bowel intussusception and does not hold the same therapeutic value in adults as it does in children [2, 20].

Surgery is the mainstay of treatment for adults with an intestinal intussusception [18]. Given the likelihood of an organic lesion serving as a lead point causing the intussusception, surgical laparotomy is almost universally the initial step in management [18, 21]. If an organic lesion such as a mass is found to be serving as an anatomical lead point for the intussusception, then surgical resection of the involved portion of bowel with or without reanastomosis is usually performed. If there is no organic lesion serving as a lead point present, then manual reduction of the affected segments of bowel is the therapeutic modality of choice [21]. Although transient and self-resolving intestinal intussusceptions have been reported in a few cases of functional intussusception from hyperglycemia, unremitting and severe abdominal pain requires surgical evaluation and immediate intervention [5, 12, 21]. In cases of functional intussusception from hyperglycemia, it is unclear whether the correction of hyperglycemia results in an immediate resolution of intestinal telescoping [5]. Clinicians should thus be weary of the possibility of intussusception in patients with severe hyperglycemia and persistent abdominal pain despite correction of metabolic derangements. Untreated or undiagnosed intussusception may result in bowel necrosis, eventual bowel perforation, and severe peritonitis, increasing the mortality from adult intussusception dramatically [2].

## 4. Conclusion

Hyperglycemia may result in intestinal dysmotility, potentially leading to intestinal intussusception in adults. In patients with severe hyperglycemia along with acidosis and hyperkalemia, intestinal intussusception should be considered in those with unremitting or worsening abdominal pain despite corrections in their previous metabolic derangements [3, 5]. Heightened suspicion of intussusception in such a patient population may lead to the appropriate diagnostic measures and prompt therapeutic interventions. 

## Figures and Tables

**Table 1:** Admission and follow-up metabolic profile after appropriate treatment with intravenous saline and insulin.

Time (hours); reference range	Serum Na* (mmol/L) (136–145)	Serum K* (mmol/L) (3.6–5.1)	Serum Cl* (mmol/L) (98–107)	Serum CO_2_* (mmol/L) (22–32)	Serum BUN* (mmol/L) (8–20)	Serum Cr* (*μ*mol/L) (0.60–1.30)	Serum glucose (mmol/L) (70–110)	Anion gap (6.0–18.0)
0**	128	5.4	87	1.0	33	2.44	1167	40
6	136	4.4	103	5	33	2.08	785	28
12	150	4	122	11	26	1.79	419	17
18	153	3.7	124	19	17	1.26	228	10
24	156	3.1	131	22	12	1.14	86	4

*Abbreviations: Na: sodium; K: potassium; Cl: chloride; CO_2_: carbon dioxide; BUN: blood urea nitrogen; Cr: creatinine.

**Note: time 0 indicates admission laboratory values.
